# The role of social identification for achieving an open-defecation free environment: A cluster-randomized, controlled trial of Community-Led Total Sanitation in Ghana

**DOI:** 10.1016/j.jenvp.2019.101360

**Published:** 2019-12

**Authors:** Miriam Harter, Nadja Contzen, Jennifer Inauen

**Affiliations:** a Eawag, Swiss Federal Institute of Aquatic Science and Technology, Dübendorf, Switzerland; b University of Groningen, Department of Psychology, the Netherlands; c University of Bern, Department of Psychology, Switzerland

**Keywords:** Community-led total sanitation (CLTS), Community social identity, Environmental sanitation, Open defecation, Ghana, Generalized Estimating Equations (GEE)

## Abstract

Unsafe sanitation practices are a major source of environmental pollution and are a leading cause of death in countries of the Global South. One of the most successful campaigns to eradicate open defecation is “Community-Led Total Sanitation” (CLTS). It aims at shifting social norms towards safe sanitation practices. However, the effectiveness of CLTS is heterogeneous. Based on social identity theory, we expect CLTS to be most effective in communities with stronger social identification, because in these communities individuals should rather follow social norms. We conducted a cluster-randomized controlled trial with 3,216 households in 132 communities in Ghana, comparing CLTS to a control arm. Self-reported open defecation rates and social identification were assessed pre-post. Generalized Estimating Equations showed that CLTS achieved lower open defecation rates compared to controls. This effect was significantly stronger for communities with stronger average social identification. The results confirm the assumptions of social identity theory. They imply that pre-existing social identification needs to be considered for planning CLTS, and strengthened beforehand if needed.

## Highlights

• CLTS intervention communities were 11 times less likely to practice open defecation at follow-up compared to controls.
• Intervention communities with higher average social identification showed lower open defecation rates.
• In control communities, higher open defecation rates were connected with higher social identificatio.
• The results highlight the need of considering the social context when planning and implementing sanitation campaigns.

## 1 Introduction

Annually, nine million people die due to environmental pollution (Landrigan et al., 2017). Unsafe sanitation, and more specifically open defecation, is one of the main causes, leading to fecal contamination of water bodies and the transmission of fecal bacteria (Prüss-Ustün et al., 2014). In 2015, 892 million people still practiced open defecation, with rates being highest in Sub-Saharan Africa (WHO & UNICEF, 2017). In Ghana, where this study is located, 31% of the rural population practiced open defecation in 2015 (WHO & UNICEF, 2017). A recent systematic review found that increasing access to safe sanitation services can reduce diarrheal diseases by 16% (Wolf et al., 2014). However, a single individual or household, by stopping open defecation, can only marginally reduce their diarrheal risk related to a fecal polluted environment (Jung, Hum, Lou, & Cheng, 2017). Research has shown that at least 75% of all households must stop open defecation to achieve a hygienically safe environment that benefits all (Clasen, Boisson et al., 2014; Jung et al., 2017; Wolf, Hunter et al., 2018). Open defecation is thus not only an individual but a collective health hazard (Geruso & Spears, 2018; Vyas, Kov, Smets, & Spears, 2016). This is comparable to other environmental challenges, such as greenhouse gas emissions, which can only be confronted if most of the population show climate-protective behavior such as reduction of individual energy consumption.

Activating social norms^1^ supporting pro-environmental behaviours helps people to act pro-environmentally (Bamberg & Möser, 2007; Steg & Vlek, 2009), such as avoiding littering in public places (Cialdini, Reno, & Kallgren, 1990), conserving household energy (Schultz, Nolan, Cialdini, Goldstein, & Griskevicius, 2007) or using safe water sources sustainably (Contzen & Marks, 2018). Similarly, activating social norms has been used in the context of sanitation (Dooley, Maule, & Gnilo, 2016). It is a key element of the behavior change campaign *Community-Led Total Sanitation (CLTS)*, which has been shown to successfully reduce open defecation by up to 33% (Pickering, Djebbari, Lopez, Coulibaly, & Alzua, 2015; Venkataramanan, Crocker, Karon, & Bartram, 2018). For Ghana, case studies on CLTS report success rates of up to 26% reduction in open defecation (Crocker et al., 2016, 2017) and scientific as well as political interest on CLTS and sanitation outcomes is steadily increasing for the Ghanaian context (Berendes et al., 2018; Nunbogu, Harter, & Mosler, 2019).

CLTS consists of a set of community-based, participatory activities, and explicitly focuses on evoking a shift towards a new social norm opposing open defecation. The influence of CLTS on social norms and thus the effect on latrine construction has already been demonstrated in research (Alemu, Kumie, Medhin, & Gasana, 2018; Harter, Mosch, & Mosler, 2018) and the consideration of social norms for the success of CLTS is gaining more attention (Dooley et al., 2016, p. 299; Novotný, Kolomazníková, & Humňalová, 2017; Venkataramanan et al., 2018). This is also true for the Ghanaian context (Osumanu, Kosoe, & Ategeeng, 2019). Because of its success in stopping open defecation, CLTS is the most widely applied sanitation campaign to date (Bongartz, Vernon, & Fox, 2016; USAID, 2018).

While randomized trials have shown that CLTS reduces open defecation compared to controls, these effects are highly heterogeneous (Harter, Inauen, & Mosler, n.d.). This means that despite the general success of CLTS, open defecation rates remain high in some communities, and the threshold of 75% households using latrines is often not reached (Crocker et al., 2016; Pickering et al., 2015; Venkataramanan et al., 2018). This indicates that inter-community differences may moderate CLTS effectiveness.

A moderator that might be at play here is social identification, defined as an individual's understanding to belong to a social group and to emotionally value the membership (Abrams & Hogg, 1990; Reynolds, Subašić, & Tindall, 2015; Tajfel, 1978). Previous research has shown that social norms particularly affect behavior in individuals strongly identified with the social group in question (e.g. (Terry, Hogg, & White, 1999; White, Smith, Terry, Greenslade, & McKimmie, 2009). One potential explanation for this effect is that strongly identified people want to be accepted and approved by their group, and may thus be eager to conform with the group's expectations, independent of whether they agree with a specific social norm or not (Abrams & Hogg, 1990; Deutsch & Gerard, 1955). Regarding CLTS, households may construct and use a latrine not because they are convinced of it, but simply because they want to be accepted in the community and therefore conform to the newly established social norm. The social identity perspective, however, proposes an alternative explanation (Tajfel & Turner, 1979; Turner, Hogg, Oakes, Reicher, & Wetherell, 1987). Self-categorization as a group member (i.e. the definition of the self in-group terms and in connection to other group members) includes a merging between group and individual; group goals become personal goals and group norms become personal norms. Accordingly, strongly identified members act in line with group norms not only because they want to conform but more so because they perceive the norm (e.g. of constructing and using latrines) as *their* personal norm, as *their* right way (Abrams & Hogg, 1990; Deutsch & Gerard, 1955).

We therefore expect that CLTS will be especially successful in reducing open defecation in communities with stronger social identification prior to CLTS implementation because people will more readily follow the newly established social norm to stop open defecation. At the individual level, we expect that people, who feel a stronger social identification than other community members, will be more likely to stop open defecation. To test our assumptions, we conducted a cluster-randomized, controlled trial, which is outlined in the following (WHO & UNICEF, 2017).

## 2 Methods

For this cluster-randomized, controlled trial, CLTS was implemented in four intervention arms and its effects on open defecation reduction were tested and compared to a control arm.^2^ Social identification prior to the intervention was tested as a moderator of CLTS effectiveness.

### 2.1 Procedures

We conducted this trial in the Northern Region of Ghana in two rural districts. In both districts we collected baseline data in February to March 2016 (for more information on the baseline survey, refer to Harter et al. (nd)). Afterwards, Global Communities, a local non-governmental organization, implemented CLTS in communities from both districts from July to November 2016.^3^ This article presents data from the long-term follow-up that was realized 14–16 months after implementation of CLTS, namely in February to March 2018 in both districts. The ethical board of the University of Zurich, Switzerland and the Ethical Review Committee of the Ghana Health Service (GHS-ERC: 05/01/2016) approved this trial.

### 2.2 Study site and clusters

The study was realized in collaboration with Global Communities and local government representatives. Global Communities selected the two districts in the Northern Region of Ghana, i.e. Bole and Sawla-Tuna-Kalba, because no CLTS campaign had been implemented there before. The local government representatives selected 132 communities within the two districts according to two eligibility criteria: accessibility (by car or motorbike due to practical reasons) and community size (minimum community size of 25 households). We grouped the communities of both districts into 25 regionally separate clusters to avoid spillover of intervention effects between close communities, and randomly allocated them to the four intervention arms (five clusters per intervention arm) and the control arm (five clusters).

### 2.3 Study participants

Trained data collectors selected study participants in the communities following the random route method (Hoffmeyer-Zlotnik, 2003, pp. 205–217). Data collectors were instructed to start from a central point of the community and interview every third household in an assigned area of the community. If no one or no eligible person was at home or if the household did not want to participate, data collectors selected the next following household. Household members were eligible if aged 18 or older and stable inhabitants of the community. If more than one household member was eligible, the participating member was selected according to their availability. We equally considered men and women, as both might take important decisions for latrine construction. Every participant gave informed written consent to participate in the study.

**Fig. 1:**
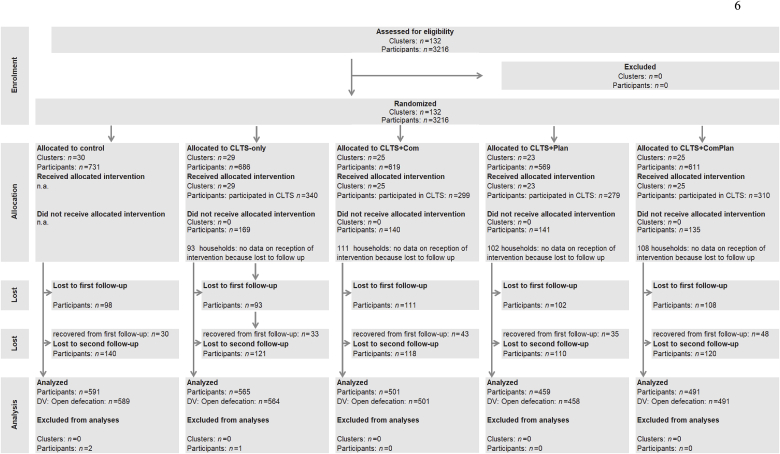
**Sample flow chart** *Note:* DV = Dependent variable. Clusters = communities, participants = interviewees within the communities. No clusters were lost to follow-up.

The sample size was calculated a priori for a cluster-randomized trial with repeated measures and a dichotomous primary outcome (Spybrook et al., 2011). Assuming an intra-cluster correlation of ρ = 0.2, 80% power, 5% α-error probability, and 20% dropout, we estimated a required sample size of 3,215 households nested in 132 communities (approx. 25 households in each) to detect a medium effect of the intervention on open defecation. For a detailed description of the sample size calculation, please refer to Harter et al. (nd). Fig. 1 displays the flow of participants through the trial.

### 2.4 Interventions

Global Communities developed intervention protocols for CLTS based on the Handbook on CLTS (Kar & Chambers, 2008). Local facilitators implemented it in three phases. First was an informative phase, where facilitators visited the community and collected information on the composition of the community and the baseline behavior. A date for a community meeting was agreed and all inhabitants were invited. The community meeting, also called triggering event, formed the second phase of CLTS. During this meeting, the facilitators motivated community members to draw a map of their community on the ground and to indicate their houses as well as the spots they used for open defecation on the map. Through asking questions about possible ways of fecal-oral transmission of pathogens, the inhabitants were expected to recognize the hygienic problems connected to open defecation. The facilitators further identified emerging leaders during the triggering event and invited them to serve as role models and to support others in the process of latrine construction. A community action plan and a date on which the community wanted to be open defecation free (ODF) was agreed. In the end of the triggering event, the facilitators explained the first step of a latrine construction, namely digging the pit and gave further information on the construction process, such as which material to use. No financial support was given to community members (including emerging leaders), however, construction materials were provided at wholesale price instead of retail prices. The third phase of CLTS included follow-up visits in the weeks after the triggering event until the community reached the status ODF, defined as at least 80% latrine coverage. During the follow-up visits, facilitators addressed any arising problems and questions regarding latrine construction. CLTS was implemented in all four intervention arms. For three of the intervention arms additional campaign activities were developed and implemented based on the Risk, Attitudes, Norms, Abilities and Self-regulation (RANAS) approach (for detailed description of implemented interventions and outcomes please refer to the intervention manual^4^ and Harter et al. (nd)). They included a household action plan and a public commitment for latrine construction. The control arm did not receive any intervention during the research phase but CLTS was implemented after the trial. In intervention communities, 72.8% (*n = *1540) of the households attended the CLTS event.

### 2.5 Data collection and outcome measures

A team of 33 local data collectors assessed outcome variables at baseline and both follow-ups. The first author, together with local personnel trained the team in a 1-week training before each of the three data collection phases. The trainings included a detailed discussion of questionnaire items and explained the correct usage of instruments and interview techniques. These were then rehearsed in role-plays. The questionnaire was translated into seven local languages as part of the data collector training of the baseline data collection, and pretested in two days and 66 interviews prior to each data collection in the field. Every interview was supervised (by research managers, interns, master students and local field supervisors), and lasted 50 min on average. Interviews included self-reported behavioral measurements, social identification, and further items on psychosocial determinants of behavior (not relevant to the present paper, for information refer to Harter et al. (nd)).

The *self-reported open defecation rates at long-term follow-up* were assessed with items based on the Safe San Index (Jenkins, Freeman, & Routray, 2014). Six items assessed the self-reported open defecation rate of each individual during the last week. The original Safe San Index includes information about all household members, whereas for this article only individual self-reported behavior at long-term follow-up was considered. Three items asked for the respondent's open defecation frequency in the mornings, middays and evenings/nights of the last week and three items asked the same for latrine use (items displayed in Supporting Information in SI Table 1). The Safe San Index represents the proportion of safely managed feces relative to total defecation instances, resulting in a range of 0–1. However, the data revealed that individuals either exclusively practiced open defecation or used a latrine. This resulted in a binary outcome variable with 0 = no open defecation and 1 = open defecation. Aggregated to community level it accounts for a communities' average open defecation rate, the proportion of people within a community who reported to practice open defecation (0–100%).

*Social identification at baseline* was measured as identification with the community on three dimensions: in-group ties, in-group affect and centrality, following items proposed by Cameron (2004). The selection of two items per dimension for this research was done in accordance with local partners, based on cultural and language considerations. Items were framed as statements with a five-point Likert-type scale for agreement. We used a visual scale with five black dots (in ascending order relative to their size) to help respondents choose one of the answer options. The data collector read out every answer option to the respondent and pointed it out on the visual scale. To test the item factor structure, we conducted an exploratory factor analysis with Principal Components Analysis and Varimax rotation with Kaiser Normalization (Field, 2009) (correlations displayed in SI Table 2 in supporting information). The factor analysis was not able to replicate the dimensions proposed by Cameron (2004), but resulted in one factor for social identification with the items of the two dimensions in-group affect and centrality loading on the factor. Whereas the items of the dimension social ties did not load on it and were therefore excluded. The remaining four items were aggregated to one scale (*M = *4.29*, SD* = 0.30, Cronbach's *α* = 0.64). Table 1 displays the four items of the scale and according descriptive measures, correlations and intra-class correlation. Aggregated at the community level, it resembles a community's average social identification.

**Table 1:** Descriptive measures and correlations for items of the social identification scale.

		n	M	SD	r		
					1	2	3
*Social identification scale*		3,216	4.28	0.30			
*Items*							
Original sub-dimension: Centrality	I often think about the fact that I am a member of this community.	3,216	4.16	1.17			
	In general, being a member of this community is an important part of my self-image.	3,216	4.29	0.99	0.42**		
Original sub-dimension: In-group Affects	In general, I am glad to be a member of this community.	3,214	4.46	0.92	0.33**	0.58**	
	I do not feel good about being a member of this community. ^a^	3,216	1.81	1.31	0.15**	0.23**	0.31**

*Note:* Items based on Cameron (2004). Items measured on a five-point Likert-scale: 1 = agree not at all to 5 = agree very much. Significance levels: **p < 0.01. *SD* = standard deviation. *r = *Pearson correlation. ^a^ question was recoded for analysis. For the social identification scale: Cronbach's α = 0.64 and P (ICC) = 0.11; ICC = Intra-class correlation.

### 2.6 Analyses

To test the moderating influence of social identification on the effect of CLTS on open defecation, we fitted a Generalized Estimating Equation (GEE) (see Zeger and Liang (1986, pp. 121–130); Zeger, Liang, and Albert (1988)) using IBM SPSS Statistics for Windows, version 24 (IBM Corp., Armonk, N.Y., USA). The model was set up using binomial distribution with logit link (Homish, Edwards, Eiden, & Leonard, 2010), because the outcome was binary. We used an exchangeable correlation structure, which assumes constant intra-cluster dependency (used for clustered data not assessed in a time-series, see Ballinger (2004)). This model accounted for the nested structure of our data with households nested in communities and further allowed the inclusion of a binary outcome (0 = no open defecation vs. 1 = open defecation). The CLTS intervention (0 = control arm; 1 = intervention arms) was entered together with the community-averaged social identification (grand-mean centered), and the individual's deviation from their community's average social identification (group-mean centering). Thereby, we were able to distinguish between community-level and individual-level effects, which may differ (Hamaker, 2012). We further added the interaction terms of the intervention with social identification at both, individual and community level, to test whether social identification at baseline moderated the intervention effect on reported open defecation at follow-up. As effect size measures, we calculated odds ratios (ORs) with asymptotic Wald 95% confidence intervals (CIs). ORs can be interpreted as increased (OR>1) or decreased (OR<1) odds of practicing open defecation for a unit increase in the predictor.

## 3 Results

### 3.1 Sample description

The respondents were on average 44.5 years old (*SD = *16.1). Slightly fewer than half were female (42%) and 21% were able to read and write. The households consisted of eight members on average (*SD = *5). In terms of religion, 26% named Islam as their religion, 49% Christianity, 19% traditional religions, and 5% mentioned to be atheists. Most of the sample reported to be farmers (80.4%) with an average monthly household income of 202 Ghanaian New Cedi (*SD = *380), equivalent to 42 USD. The households of the sample therefore lay on average below the poverty line proposed by the World Bank of 57 USD per individual per month (Atkinson, 2017).

### 3.2 Randomization check and dropout analysis

Table 2 shows baseline characteristics for intervention and control arms. Chi-Square tests and variance analysis revealed that the groups significantly differed on all characteristics except for age, household size and number of dropouts, which were equally distributed. At baseline, 89.9% of the control and 97.2% of the intervention arm reported to practice open defecation. The main analyses reported in this paper were rerun and characteristics were included that had shown significant differences between intervention and control group at baseline. Even though the effect sizes were small (Cohen, 1992; Ferguson, 2009; Trusty, Thompson, & Petrocelli, 2004), those characteristics were included in sensitivity analyses as they were considered to be potential confounding variables.

**Table 2:** Baseline sample characteristics for intervention and control arms.

*n*	Control Group	Intervention	*Cramer's V*	*p*	
	740	2476			
**Occupation**			0.188	<.001	
Farming	66.3%	84.5%			
Other (trading, mining, fishing)	33.7%	15.5%			
**Religion**			0.193	<.001	
Islam	39.4%	22.1%			
Christian	43.5%	51.0%			
Traditional religion	13.4%	20.9%			
Atheists	3.6%	6.0%			
**Female respondents**	50.4%	40.2%	0.087	<.001	
**Ability to write**	25.1%	19.8%	0.055	.002	
**Dropout**	80.4%	81.3%	0.009	.603	

**Open defecation rate**	89.9%	97.2%	0.148	<.001	
	*M (SD)*	*M (SD)*	*F*	*p*	*d*
**Age**	44.39 *(16.30)*	44.58 *(16.08)*	0.06	.805	0.01
**Income**	268.65 *(530.55)*	183.21 *(320.16)*	28.13	<.001	−0.22
**Household size**	8.42 *(4.63)*	8.80 *(4.92)*	3.30	.069	0.08
**Social identification**	4.24 *(0.80)*	4.28 *(0.76)*	4.79	.029	0.04

*Note:* Effect sizes for independent means according to Cohen (1992): *d = *0*.*2 (small), *d = 0.5* (medium), *d = *0*.*8 (large) and for Cramer's *V*: *V* = 0.1 (small), *V* = 0.3 (medium), *V* = 0.5 (large) (Ferguson, 2009).

Furthermore, we compared respondents who participated in both panel surveys (*n = *2,607) to respondents only participating in the baseline survey (dropouts, *n = *609, 18.9%) on the same characteristics. Chi-square tests and variance analyses showed that the study dropouts were significantly less socially identified with their community, less likely to be farmers, had a higher probability for literacy, were significantly younger and had a higher income compared to analyzed participants. Open defecation rates were not significantly different between study dropouts and participants remaining in the sample (see SI Table 3 in Supporting Information).

### 3.3 Intervention effects on open defecation and the influence of social identification

In the CLTS intervention arms, 46.4% (*SD = *49.9%) of the individuals reported to practice open defecation at follow-up, compared to 88.4% (*SD = *32.0%) in the control arm. As indicated by the GEE model results (see Table 3), the OR for the intervention group indicates that intervention participants were 11 times less likely to practice open defecation at follow-up than controls (*Β[SE]* = −2.42 [0.33]*, OR* = 0.09*, p* < 0.001).

**Table 3:** Parameter estimates for Generalized Estimating Equation of intervention main effects and interaction effects with social identification on open defecation at follow-up.

	*B (SE)*	*p*	*OR*	95% Wald Confidence Interval for OR	
				LL	UL
(Intercept)	0.24 *(0.16)*	0.145	1.27	0.92	1.74
Effect of CLTS compared to control arm^a^	−2.42 *(0.33)*	<0.001	0.09	0.05	0.17
Effect of individual social identification in control arm	0.25 *(0.24)*	0.305	1.28	0.80	2.06
Effect of community's average social identification in control communities	7.06 *(2.28)*	0.002	1169.42	13.52	101186.42
Interaction effect of individual social identification with CLTS	−0.65 *(1.05)*	0.534	0.52	0.07	4.08
Interaction effect of community's average social identification with CLTS	−11.70 *(4.15)*	0.005	<0.01	<0.01	0.03

*Note: N* = 2606, *B* = unstandardized regression coefficients. *SE* = Standard error. *OR* = Odds ratio. *LL = *Lower level, *UL* = Upper level. Probability distribution: binomial, link function: logit. All *p*-values are two-tailed. Outcome (self-reported): 0 = no open defecation, 1 = open defecation. Social identification was group-mean centered (individual) and grand-mean centered (community level). ^a^ CLTS: 0 = control arm, 1 = CLTS interventions.

**Fig. 2:**
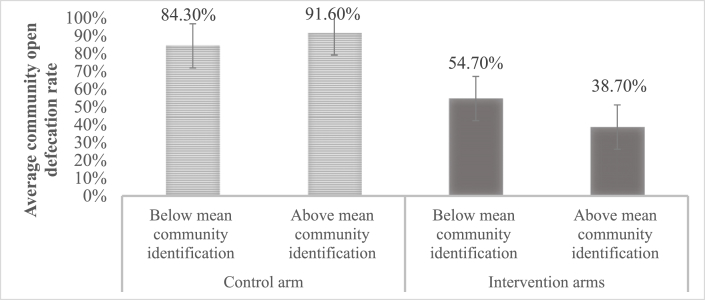
Average community open defecation rates in control and intervention arm depending on community's average social identification. Reported average community open defecation rate for the control arm (light grey and dashed) and intervention arms (dark grey and solid).

Fig. 2 shows the community-averaged open defecation rate in control and intervention arms moderated by community's average social identification. In line with our hypothesis, CLTS intervention communities with stronger community-averaged social identification reported less open defecation at follow-up than those with lower community-averaged social identification (*Β[SE]* = −11.70 [4.15], *p* = 0.005). The control arm showed opposite effects: communities with stronger community-averaged social identification reported higher open defecation rates at follow-up than those with lower community-averaged social identification (*Β[SE]* = 7.06 [2.28]*, p* = 0.002). In both, the control and intervention arms, the effects of individuals' social identification pointed in the same direction as the community-averaged social identification, but were not significant (control arm: *Β[SE]* = 0.25 [0.24], *p* = 0.305; intervention arm: *Β[SE]* = −0.65 [1.05]*, p* = 0.534). Sensitivity analyses revealed that including the baseline characteristics, and adjusting for baseline behavior, did not substantively change the findings. Only age and literacy had significant but small reducing effects on open defecation (age: *Β[SE]* = −0.01 [<0.01], *p* = 0.002; literacy: *Β[SE]* = −0.27 [0.10]*, p* = 0.008).

## 4 Discussion

This study corroborated previous findings that CLTS is an effective intervention to reduce open defecation. In our sample, at the long-term follow-up, 53.6% of individuals in the intervention arms did not defecate in the open anymore. While this rate is still behind the threshold of 75% of all community members that would need to stop open defecation to reach an incremental health benefit at community level (Jung et al., 2017; Wolf et al., 2018), it is comparable to most randomized trials of CLTS. A recent review on CLTS reports that the majority of interventions achieve around 50–80% rates of stopping open defecation (USAID, 2018).

That the reported rate in our study is at the lower end, can partly be explained by the short time elapsed between intervention and follow-up survey. At the time of the survey, many latrines (61.6%) were still under construction and therefore still not in use. In the intervention arm, the figure of 46.4% of the respondents that reported to practice open defecation, might likely decrease as soon as the construction process of the remaining latrines is completed. We expect this, because in our sample the clear majority of households that owned a completed latrine also used it (93.6%). This is surprising when compared to previous research on latrine ownership and use, for example from India, where it was found that only 47% of the owned latrines were actually used (Barnard et al., 2013).

More importantly, this study showed for the first time that social identification within communities moderates the effectiveness of CLTS on open defecation. Specifically, our hypothesis regarding the influence of social identification on the intervention effects was supported: CLTS was more successful in communities with stronger social identification prior to the intervention. In communities with stronger community-averaged social identification, 38.7% of respondents reported to practice open defecation, compared to 54.7% in communities with lower average social identification. Our findings extend previous findings of a randomized trial on CLTS in Indonesia on the importance of communities’ pre-existing social conditions for intervention success (Cameron, Olivia, & Shah, 2015). The researchers were able to show in a randomized trial, that communities with higher initial social capital, i.e., higher trust and cohesion, were more likely to have higher latrine coverages.

We suppose that the reported moderating effect of social identification on CLTS effectiveness work through an increase in new social norms that oppose open defecation. In communities in which individuals strongly identify with their community, individuals wish to conform to the new norm, which will lead to better CLTS outcomes (e.g. Terry et al. (1999) and White et al. (2009)). Alternatively, and based on social identity theory, strongly identified individuals might not only conform to the social norms, but internalize them as their own goal and their personal norms (Abrams & Hogg, 1990; Deutsch & Gerard, 1955). Future research can test these assumed mechanisms of CLTS and disentangle whether CLTS truly evokes a shift in social norms and whether these translate, moderated by social identification, into personal norms.

Interestingly, our data showed opposite effects in control communities: higher open defecation rates were reported in communities with stronger compared to communities with weaker average social identification. This might be because in communities without CLTS intervention the prevailing social norms were supporting open defecation, as no impulse of change had occurred. This finding supports social identity theory; communities with stronger community-averaged social identification follow, or better said incorporate, the prevailing social norms, whether the social norms suggest stopping open defecation – as in intervention communities – or the opposite – as in control communities (Abrams & Hogg, 1990; Cialdini et al., 1990). Schultz et al. (2007) described this effect of salient norms that lead to an undesired behavior as *“the destructive potential of social norms” (p.431)*. A departure from prevailing social norms, such as stopping open defecation when open defecation is what the rest of the community members are doing, may only be possible for community contexts where social identification is weak, i.e., where community members do not define themselves through their community and are thus less inclined to follow the prevailing social norms (Abrams & Hogg, 1990; Deutsch & Gerard, 1955).

Finally, our results did not suggest any additional effects of social identification at the individual level, over and above the community-level effects. It seems that the moderating effect of social identification is a truly community-based phenomenon.

To sum up, our results highlight the importance of social identification especially for collective environmental challenges, such as open defecation. This is in line with the increasing interest of the CLTS community to consider the social context in CLTS planning and implementation (Dooley et al., 2016, p. 299; Novotný et al., 2017; USAID, 2018).

For the implementation practice, this means that communities with strong social identification provide a fertile ground for CLTS implementation. To improve CLTS planning, we therefore suggest the assessment of social identification in a first step. If social identification is found to be weak, activities should be carried out to foster social identification prior to CLTS implementation, as has been recommended for the field of collective action (Van Zomeren, Postmes, & Spears, 2008). Such activities might include enabling interaction between community members (Jans, Leach, Garcia, & Postmes, 2015) or directing attention to neighboring communities that have already eliminated open defecation, for example forming a competition-like situation and pointing out the differences to an out-group (Jans, Bouman, & Fielding, 2018; Tajfel & Forgas, 2000, pp. 49–63). In cases where social identification cannot be strengthened before a CLTS implementation, by-laws or sanctions for people not following the norms might be enforced, which is proposed by the CLTS Handbook (Kar & Chambers, 2008) and in social psychology literature to solve social dilemma situations (e.g. (De Cremer, Hoogervorst, & Desmet, 2012)). The control group findings imply that communities with strong social identification are potentially at risk of increasing or reinforcing open defecation practices. These communities should therefore be selected with high priority for sanitation interventions to avoid such tendencies, and leverage promising responses to interventions due to strong identification.

### 4.1 Strengths and limitations

To the best of our knowledge, this study is the first that investigated the influence of social identification on the effect of CLTS on open defecation. It was fully powered with a sample of 2,606 households for the longtime follow-up survey and 3,216 households in the baseline. With 132 communities, it allowed analysis at the community level, and the investigation of the deviation of individuals from community means. CLTS was implemented under real conditions in rural Ghana in a variety of local contexts, such as different community sizes and ethnical compositions. This allows assuming high external validity.

The study, however, has the following limitations. The first relates to the causal relationship of social identification moderating CLTS intervention effects. Because we did not experimentally manipulate social identification, the found moderating effect could be attributable to other influencing factors, for example community size or heterogeneity within communities. Future research should manipulate the strength of social identification to provide further evidence for the presented moderating effects in this article.

Open defecation was assessed through self-reports. However, strengthening the validity of the self-report measure, the use of latrines was verified by observation of enumerators,^5^ which correlated strongly with the self-reported behavior (*r*^*2*^ = 0.72, *p* < 0.001).

The scope of this study did not allow for assessment of open defecation rates more than one year, including participants that might have reverted to open defecation. Long-term change should be included in future research.

Social identification was measured using six items only and for analysis, only four items were included. However, the scale showed relatively low reliability (Cronbach's α = 0.64). Furthermore, the six items used for the social identification scale applied in this article were not able to replicate the three dimensions of social identification postulated by Cameron (2004). The reason for this may be that only two items per dimension were included to keep the questionnaire as brief as possible to minimize participant burden. Future studies should use more items to allow for a more detailed consideration of social identification dimensions.

## 5 Conclusion

This study reports the success of CLTS on reducing open defecation rates and highlights the relevance of including social conditions into planning of sanitation campaigns, such as CLTS. Specifically, the consideration of communities’ social identification is crucial for the success of CLTS on reducing open defecation, as it might be able to intensify the effects of the intervention. We therefore recommend to assess the level of social identification within target communities and plan CLTS interventions accordingly, meaning to strengthen, if needed, the social identification among community members before a sanitation intervention. Further, this is the first time that the concept of social identification was studied in environmental sanitation and points to the potential influence of social identification in other water and sanitation related behaviors in low- and middle-income countries.

## Footnotes

1 We refer to the definition of social norms provided by Cialdini and Trost (1998): “rules and standards that are understood by members of a group, and that guide/constrain social behavior without the force of laws” (page 152). Additionally, we distinguish social norms further “to the extent to which they are injunctive, prescribing the valued social behavior, versus descriptive, informing us about how others act in similar situations” (page 152).

2 Four different variations of CLTS were implemented but no differences in intervention effects were found between intervention arms Harter et al. (nd). Therefore, the four arms were combined and compared in one group against the control group.

3 A mid-term follow-up survey was conducted in both districts four to six months after implementation, namely in February to March 2017 (the effects are reported in Harter et al. (nd)).

4 The intervention manual is accessible online: https://osf.io/gdcqs/?view_only=eb1238cbaebf403c8618f971e500c206.

5 Enumerators did short observational spot-checks on the latrines and assessed the level of construction and signs of use, such as cleansing material.
