# Supplementary material for: The role of social identification for achieving an open-defecation free environment: A cluster-randomized, controlled trial of Community-Led Total Sanitation in Ghana
Source: J Environ Psychol. 2019 Dec;66:101360. doi: 10.1016/j.jenvp.2019.101360 (PMC6919339; doi:10.1016/j.jenvp.2019.101360)
Supplement: Multimedia component 1 [file mmc1.docx]

**Supporting information.**

**SI** **Table 1.** Behavioral assessment of open defecation frequency

| Introduction to defecation behavior measurement | We would like to know some things about your personal defecation habit. Please try to answer each question as precisely as you can. The following questions refer to situations, when you are in your community. |
| --- | --- |
| Open defecation frequency | On how many of the last 7 mornings did you defecate in the open? (e.g., field, bush, roadside, side of canal, back of house, etc.). *0= no days to 7= every day* |
|  | On how many of the last 7 middays did you defecate in the open? (e.g., field, bush, roadside, side of canal, back of house, etc.). *0= no days to 7= every day* |
|  | On how many of the last 7 evenings or nights did you defecate in the open? (e.g., field, bush, roadside, side of canal, back of house, etc.). *0= no days to 7= every day* |
| Latrine use frequency | On how many of the last 7 mornings did you use your latrine? *0= no days to 7= every day* |
|  | On how many of the last 7 middays did you use your latrine? *0= no days to 7= every day* |
|  | On how many of the last 7 evenings or nights did you use your latrine? *0= no days to 7= every day* |

*Note.* Items based on the Safe San Index (Jenkins et al., 2014)

**SI Table 2.** Correlation of social identification items

|  |  | 1 | 2 | 3 | 4 | 5 |
| --- | --- | --- | --- | --- | --- | --- |
| In-group Ties | I have a lot in common with other community members. |  |  |  |  |  |
|  | I find it difficult to form a bond with other community members. | 0.06** |  |  |  |  |
| Centrality | I often think about the fact that I am a member of this community. | 0.19** | 0.09** |  |  |  |
|  | In general, being a member of this community is an important part of my self-image. | 0.24** | 0.11** | 0.42** |  |  |
| In-group Affects | In general, I am glad to be a member of this community. | 0.23** | 0.07** | 0.33** | 0.58** |  |
|  | I do not feel good about being a member of this community. | -0.01 | 0.15** | 0.15** | 0.23** | 0.31** |

Significance levels: ***p* < 0.01. Answer scale: 1= agree not at all to 5= agree very much.

**SI Table 3.** Descriptive baseline measures for dropouts and respondents remaining in the sample

|  | Dropouts | Respondents | *Cramer's V* | *p* |  |
| --- | --- | --- | --- | --- | --- |
| *n* | 609 | 2607 |  |  |  |
| Occupation |  |  | 0.05 | 0.003 |  |
| farming | 76.1% | 81.3% |  |  |  |
| other (trading, mining, fishing) | 23.9% | 18.7% |  |  |  |
| Religion |  |  | 0.04 | 0.271 |  |
| Islam | 26.9% | 26.1% |  |  |  |
| Christian | 53.1% | 48.8% |  |  |  |
| Traditional religion | 15.4% | 19.6% |  |  |  |
| Atheists | 4.5% | 5.5% |  |  |  |
| Female respondents | 43.3% | 42.4% | 0.01 | 0.665 |  |
| Ability to write | 30.2% | 18.9% | 0.11 | <.001 |  |
| Open defecation | 95.3% | 95.6% | <0.01 | 0.802 |  |
|  |  |  |  |  |  |
|  | Mean *(SD)* | Mean *(SD)* | F | *p* | *d* |
| Age | 39.27 *(17.48)* | 45.15 *(15.85)* | 34.16 | <.001 | 0.26 |
| Income | 256.53 *(511.92)* | 189.68 *(341.52)* | 15.20 | <.001 | -0.18 |
| Household size | 8.36 *(4.85)* | 8.78 *(4.85)* | 3.66 | 0.056 | 0.09 |
| Social identification | 4.17 *(0.84)* | 4.30 *(0.75)* | 7.21 | 0.007 | 0.16 |

*Note:* Effect sizes for independent means according to Cohen (1992): *d*=.2 (small), *d*=.5 (medium), *d*=.8 (large) and for Cramer’s *V*: *V*=.1 (small), *V*=.3 (medium), *V*=.5 (large) (Ferguson, 2009). Reasons for drop-out: 68.1% (*n*=415) had currently travelled or left the community, 12.9% (*n=*78) passed away.
